# Triptolide Suppresses Glomerular Mesangial Cell Proliferation in Diabetic Nephropathy Is Associated with Inhibition of PDK1/Akt/mTOR Pathway: Erratum

**DOI:** 10.7150/ijbs.53769

**Published:** 2020-10-03

**Authors:** Fei Han, Mei Xue, Yunpeng Chang, Xiaoyu Li, Yang Yang, Bei Sun, Liming Chen

**Affiliations:** Key Laboratory of Hormones and Development (Ministry of Health), Tianjin Key Laboratory of Metabolic Diseases, Tianjin Metabolic Diseases Hospital & Tianjin Institute of Endocrinology, Tianjin Medical University

In our paper 1, when we were preparing the blot picture of p-mTOR in Figure 6D, we mistakenly copied the above blot picture of PDK1 which were also in Figure 6D, and the original picture of p-mTOR was omitted. We would like to apologize for any inconvenience caused to the readers by these changes. We repeated the western blot assay and confirmed that the results were in accordance with the published results. It is important to state that this correction do not affect our study's results in the published paper. The original pictures of PDK1 and p-mTOR were attached as supplementary figure 1. Figure 6D should be corrected as follows.

## Supplementary Material

Supplementary figure.

## Figures and Tables

**Figure 6:**
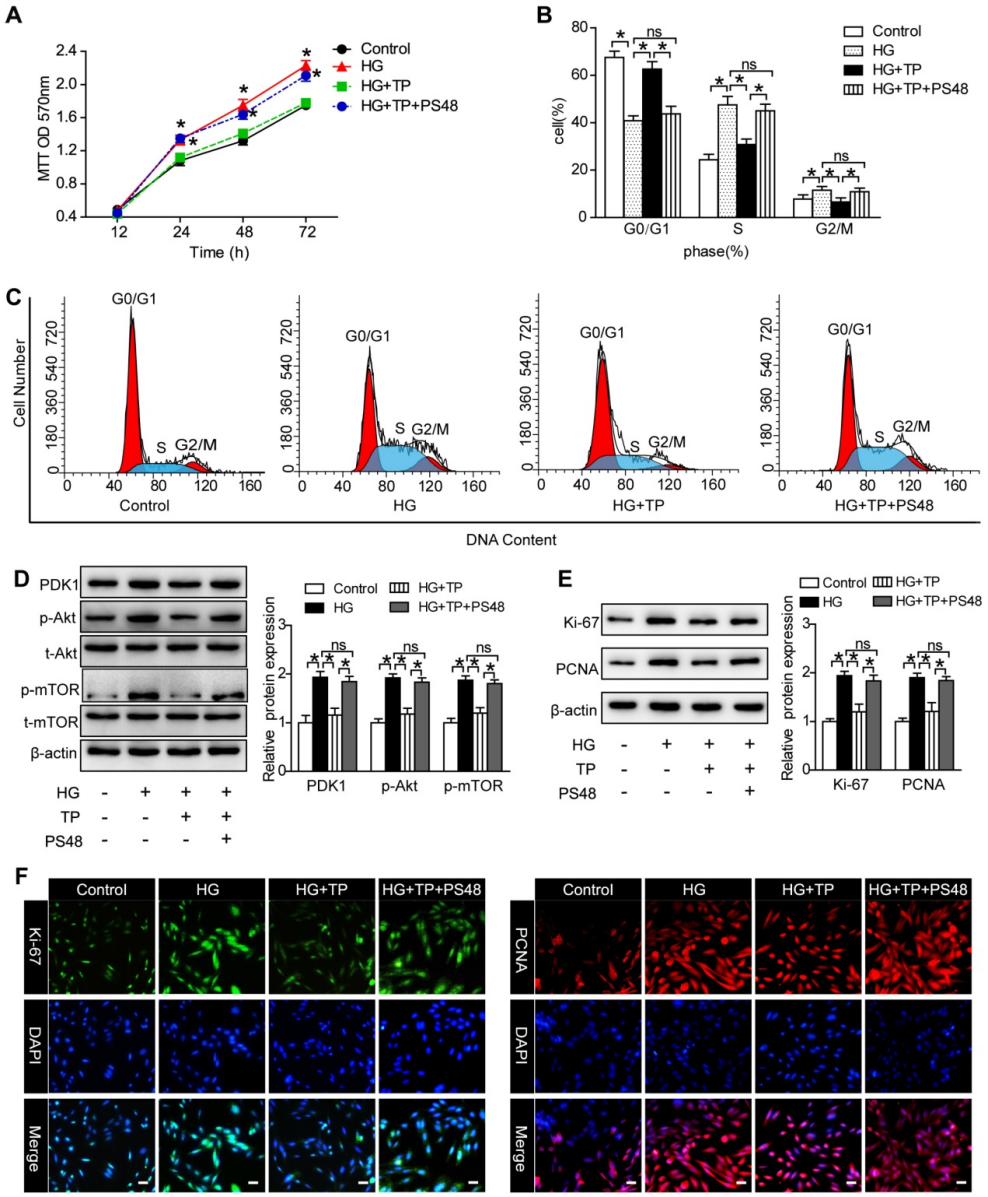
** PDK1 activator reversed the effect of TP on mesangial cell proliferation.** (A) MTT assay in the cells treated with HG for different times. *P < 0.05 vs. Control group, #*P* < 0.05 vs. HG+TP group. (B and C) Flow cytometry analysis of cell cycle in the HRMCs treated for 72 h. (D) Protein expression of PDK1/Akt/mTOR pathway in HRMCs treated for 72 h. (E) Protein expression of Ki-67 and PCNA in HRMCs treated for 72 h. (F) Immunofluorescence images of Ki-67 and PCNA. The scale bar represents 10 μm. Data were reported as mean ± S.D.. *P < 0.05; ns represents no significance

## References

[1] Han F, Xue M, Chang YP, Li XY, Yang Y, Sun B, Chen LM (2017). Triptolide Suppresses Glomerular Mesangial Cell Proliferation in Diabetic Nephropathy Is Associated with Inhibition of PDK1/Akt/mTOR Pathway. International Journal of Biological Sciences.

