# Supplementary material for: Triptolide Suppresses Glomerular Mesangial Cell Proliferation in Diabetic Nephropathy Is Associated with Inhibition of PDK1/Akt/mTOR Pathway: Erratum
Source: Int J Biol Sci. 2020 Oct 3;16(15):3037–8. doi: 10.7150/ijbs.53769 (PMC7545705; doi:10.7150/ijbs.53769)
Supplement: Supplementary file 1 — Supplementary figure. [file ijbsv16p3037s1.pdf]

Supplementary figure 1

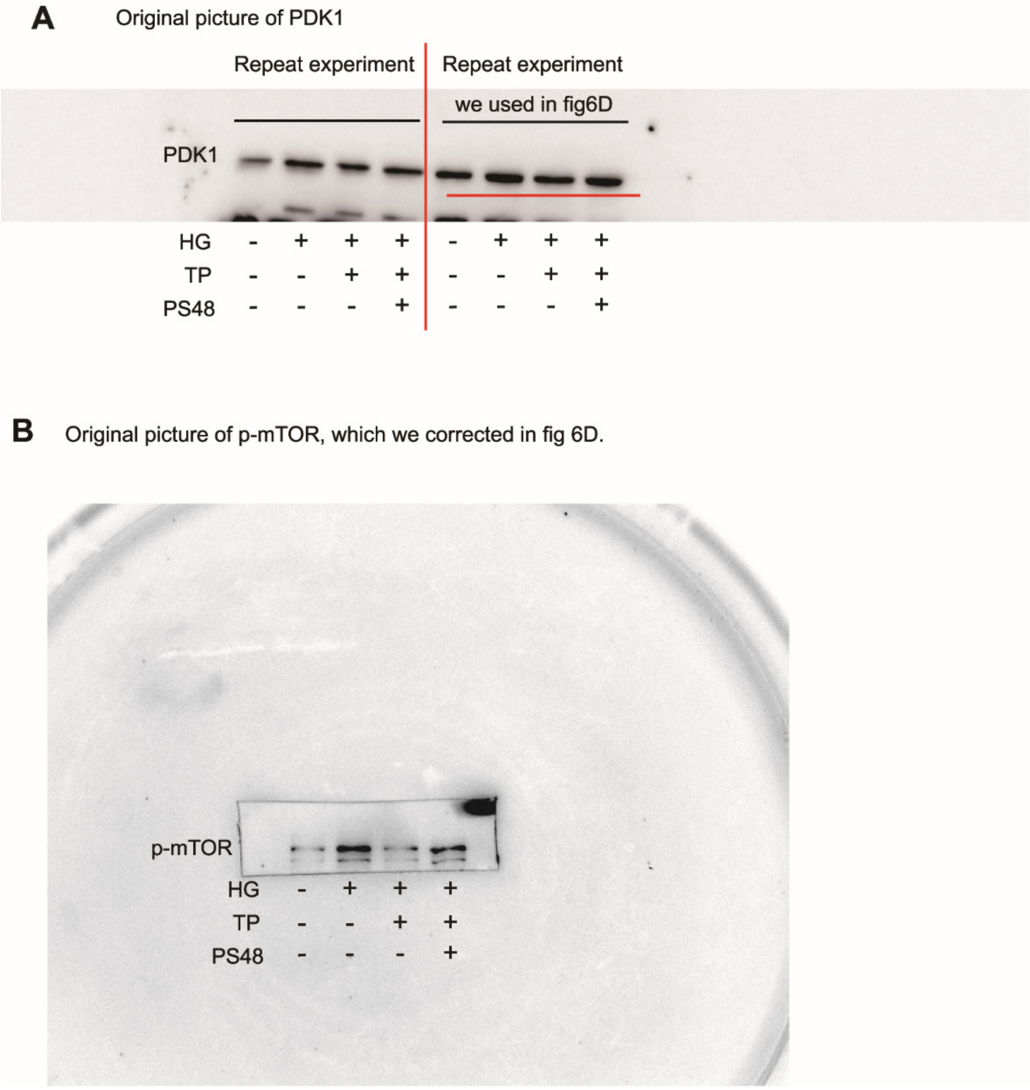

Supplementary figure 1. (A) The original blot picture of PDK1. We repeated the experiment on the same gel, and the results were similar. The second group was the exact picture that we presented in the paper. (B) The original blot picture of p-mTOR.
